# Competitive Repression of the *artPIQM* Operon for Arginine and Ornithine Transport by Arginine Repressor and Leucine-Responsive Regulatory Protein in *Escherichia coli*

**DOI:** 10.3389/fmicb.2019.01563

**Published:** 2019-07-12

**Authors:** Oscar E. Torres Montaguth, Indra Bervoets, Eveline Peeters, Daniel Charlier

**Affiliations:** Research Group of Microbiology, Department of Bioengineering Sciences, Vrije Universiteit Brussel, Brussels, Belgium

**Keywords:** ArgR, Lrp, arginine transport, ornithine, *Escherichia coli*, reporter gene assays, protein-DNA interactions, transcription factors

## Abstract

Two out of the three major uptake systems for arginine in *Escherichia coli* are encoded by the *artJ*-*artPIQM* gene cluster. ArtJ is the high-affinity periplasmic arginine-specific binding protein (ArgBP-I), whereas *artI* encodes the arginine and ornithine periplasmic binding protein (AO). Both ArtJ and ArtI are supposed to combine with the inner membrane-associated ArtQMP_2_ transport complex of the ATP-binding cassette-type (ABC). Transcription of *artJ* is repressed by arginine repressor (ArgR) and the *artPIQM* operon is regulated by the transcriptional regulators ArgR and Leucine-responsive regulatory protein (Lrp). Whereas repression by ArgR requires arginine as corepressor, repression of P*_artP_* by Lrp is partially counteracted by leucine, its major effector molecule. We demonstrate that binding of dimeric Lrp to the *artP* control region generates four complexes with a distinct migration velocity, and that leucine has an effect on both global binding affinity and cooperativity in the binding. We identify the binding sites for Lrp in the *artP* control region, reveal interferences in the binding of ArgR and Lrp *in vitro* and demonstrate that the two transcription factors act as competitive repressors *in vivo*, each one being a more potent regulator in the absence of the other. This competitive behavior may be explained by the partial steric overlap of their respective binding sites. Furthermore, we demonstrate ArgR binding to an unusual position in the control region of the *lrp* gene, downstream of the transcription initiation site. From this unusual position for an ArgR-specific operator, ArgR has little direct effect on *lrp* expression, but interferes with the negative leucine-sensitive autoregulation exerted by Lrp. Direct arginine and ArgR-dependent repression of *lrp* could be observed with a 25-bp deletion mutant, in which the ArgR binding site was artificially moved to a position immediately downstream of the *lrp* transcription initiation site. This finding is reminiscent of a previous observation made for the *carAB* operon encoding carbamoylphosphate synthase, where ArgR bound in overlap with the downstream promoter P2 does not block transcription initiated 67 bp upstream at the P1 promoter, and further supports the hypothesis that ArgR does not act as an efficient roadblock.

## Introduction

**Figure 1:**
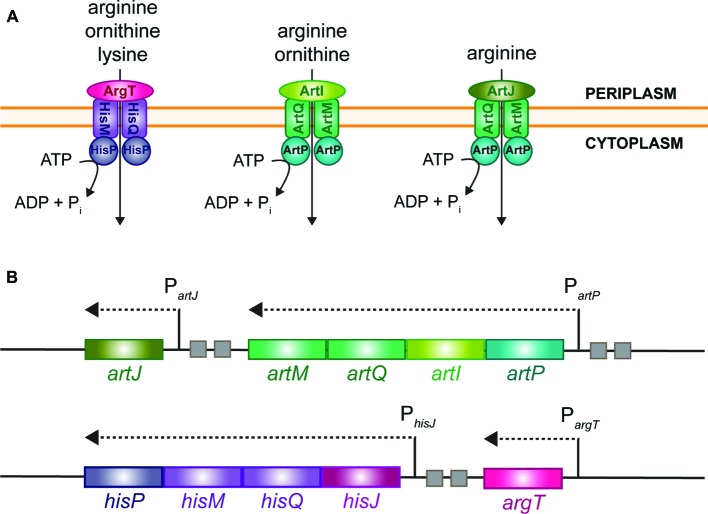
Schematic representation of the different import systems for arginine in *E. coli* **(A)** and organization of the cognate gene clusters on the chromosome **(B)**. Small squares represent ARG boxes.

Arginine is a particularly important amino acid for bacterial cell growth, physiology, and survival in stress conditions. It is not only essential for protein synthesis, but plays an equally important role in extreme acid resistance and pH homeostasis, is a precursor for the biosynthesis of the polyamines spermidine and spermine, and may serve as a source of nitrogen, carbon, and energy upon degradation *via* distinct catabolic routes (Charlier and Glansdorff, 2004; Reitzer, 2005; Leroy and Charlier, 2017). Hence, the biosynthesis, catabolism, uptake, and export of arginine are tightly regulated. Here, we focus on regulation of arginine uptake in *Escherichia coli* K-12 that possesses several transport systems of the ATP-binding cassette-type (ABC) for the import of arginine (Figure 1A), which is energetically more favorable than its biosynthesis (Saier, 2000; Hosie and Poole, 2001; Burkovski and Krämer, 2002; Davidson and Chen, 2004; Biemans-Oldehinkel et al., 2006). These various import systems are encoded by two gene clusters, *artPIQM*-*artJ* and *argT*-*hisJQMP* (Figure 1B). They differ in substrate specificity and affinity for arginine, and exhibit differences in the regulation of synthesis and activity (for an overview, see Caldara et al., 2007). These differences indicate that distinct transport mechanisms may be active in different growth conditions and fulfill different physiological needs. Each gene cluster encodes two periplasmic binding proteins, ArtJ and ArtI and HisJ and ArgT, respectively. ArtJ (alias ArgBP-I) is the arginine-specific binding protein, and has the highest affinity for arginine (*K*_d_ 0.4 μM) (Rosen, 1971; Celis, 1981). ArtI binds arginine and its precursor ornithine (Wissenbach et al., 1993, 1995), ArgT (LAO) binds the basic amino acids lysine, arginine (*K*_d_ 1.5 μM), and ornithine (Celis et al., 1973; Rosen, 1973), whereas HisJ binds histidine (*K*_d_ 0.11 μM) and with low affinity also arginine (*K*_d_ 10 μM) (Kustu and Ames, 1973). HisJ and ArgT, which share 70% amino acid sequence identity, associate with the HisQMP_2_ complex consisting of the integral membrane proteins, HisQ and HisM, and two membrane-associated HisP subunits that bind ATP and provide the energy for active transport (Kerppola et al., 1991; Ames et al., 2001). In analogy, ArtJ and ArtI are supposed to associate with the ArtQMP_2_ complex that is similar to HisQMP_2_.

In exponentially growing cells, the *artJ*, *artPIQM,* and *hisJQMP* gene and operons are transcribed from a σ^70^-dependent promoter and regulated by ArgR (Wissenbach et al., 1995; Caldara et al., 2007), the master regulator of arginine biosynthesis genes and operons (Charlier et al., 1992; Tian et al., 1992; Wang et al., 1998). In contrast, *argT* is transcribed from a σ^54^-dependent promoter that is activated by the transcriptional regulator NtrC in conditions of nitrogen limitation, but is apparently not regulated by ArgR (Schmitz et al., 1988; Barrios et al., 1999; Reitzer, 2005; Cho et al., 2011, 2015).

Previously, we have shown that hexameric arginine-bound ArgR binds to two 18 bp semi-palindromic ARG boxes separated by 3 bp, the canonical design of an ArgR binding site in *E. coli* (Charlier et al., 1992) in the *artJ*, *artP,* and *hisJ* control regions (Caldara et al., 2007). These ArgR binding sites overlap the −35 promoter element of *artJ* and *artP* to the same extent, but are located slightly more upstream in the *hisJ* control region. Furthermore, *in vitro* binding studies and single-round *in vitro* transcription assays indicated that arginine-bound ArgR inhibits transcription initiation at P*_artJ_* through direct steric exclusion of RNA polymerase binding. This is not the case at P*_hisJ_*, where inhibition is less pronounced and repression relies on a different mechanism, likely involving ArgR-induced DNA conformational changes (Caldara et al., 2007).

Genome-wide expression profiling studies, chromatin immunoprecipitation (ChIP-chip), and genomic systematic evolution of ligands by exponential enrichment (SELEX) data indicated that transcription of the *artPIQM* operon is also down-regulated by the global transcription regulator leucine-responsive regulatory protein (Lrp) in a leucine-sensitive manner (Hung et al., 2002; Cho et al., 2008; Shimada et al., 2015). Furthermore, on the basis of high-throughput sequencing of exonuclease-treated chromatin-immunoprecipitated DNA (ChIP-exo), it was suggested that ArgR binds to the *lrp* control region and interferes with Lrp-mediated regulation by directly affecting the synthesis of this global regulator (Cho et al., 2011, 2015). In spite of these recent developments, the molecular mechanism of Lrp-mediated control of *artP* expression and its potential interplay with ArgR in the control of arginine transport remain poorly documented. Furthermore, even though ArgR effectively binds to the long *lrp* leader region, as also confirmed and analyzed in greater detail in this work, the observed effect on *lrp* gene expression was small, and the unraveling of the underlying molecular mechanism requires further investigation.

Here, we present an in depth molecular analysis of the effects of the transcriptional regulators ArgR and Lrp on transcription initiation at both P*_artP_* and P*_lrp_*. To reach this goal, we perform *in vivo* reporter gene assays in various isogenic backgrounds (WT, ∆*argR*, *lrp*::Tn*10* and double ∆*argR lrp*::Tn*10* mutants) and *in vitro* DNA binding assays with purified ArgR and Lrp, alone and in combination. This allowed us to determine the regulatory effects of both transcription factors, to delimit their precise binding sites, to evaluate the effects of ligands on both *in vivo* regulation and *in vitro* DNA binding, and to reveal potential interferences in the binding and action of the two transcription factors.

## Materials and Methods

### *E. coli* Strains and Plasmid Constructions

DH5α [F^−^ Φ80*lacZ*∆M15 ∆(*lacZYA*-*argF*)U169 *hsd*R17U (rK^−^ mK^+^) *phoA supE*44 *recA*1 *endA*1 *gyrA*96 *thi*-1 *relA*1] was used for cloning purposes. Strains CSH100 (F′), FW102 (F^−^, Sm^r^) and its derivatives FW102 *lrp*::Tn*10* and FW102 ∆*argR* have been described (Whipple, 1998; Devroede et al., 2004; Peeters et al., 2009). The double mutant FW102 ∆*argR lrp*::*Tn*10 was constructed by P1vir-mediated generalized transduction of FW102 ∆*argR* with a phage lysate prepared on the single *lrp*::Tn*10* derivative of strain FW102 and selection of tetracycline-resistant transductants. Plasmid pFW11-null (Km^r^, Cm^r^) (Whipple, 1998) and its derivative pFW-p/o-*hisJ* carrying the P*_hisJ_*-*lacZ* fusion (Caldara et al., 2006) and pFW-p/o-*artJ* carrying the P*_artJ_*-*lacZ* fusion (Caldara et al., 2007) have been described. Plasmids pFW-p/o-*artP* and pFW-p/o-*lrp* carrying the P*_artP_*-*lacZ* and P*_lrp_*-*lacZ* fusion, respectively, were constructed by PCR amplification of the control region of *artP* (222 bp fragment from −195 to +26) and *lrp* (369 bp fragment from −118 to +250) with genomic DNA of strain FW102 as template and the oligonucleotide pairs DC526f (5′-GGAATTCCGAGAATCGCTAACGACTTG-3′) plus DC392r (5′-CGGGATCCCGTATACTGGCAGTCTGATAGC-3′) and DC1464f (5′-CGGAATTCGCTTTATAAGCCGATTAAATGATG-3′) plus DC1465r (5′-CGGGATCCGTATTCCTTCCCTACTCCTGTC-3′) as primers, and ligation of the EcoRI and BamHI digested amplicon in similarly digested and dephosphorylated pFW11-null plasmid DNA. The 25-bp deletion mutant derivative (∆25) of the P*_lrp_*-*lacZ* construct was obtained by the overlap extension mutagenesis method (Higuchi et al., 1988) with the mutagenic primers DC1562f (5′-TGCGCATAACCATGCATGTAAATACC-3′) and DC1561r (5′-GTATTTACATGCATGGTTATGCGCACTCGAATGTTTTCGCAAAACACCAG-3′). Recombinant plasmids were transformed in strain CSH100 and double cross-over events transferring the promoter-operator-*lacZ* fusions to the single copy F′-episome were obtained by conjugation with the F^−^ strain FW102 and its single and double ∆*argR* and *lrp*::Tn*10* derivatives, and selection of Km^r^ and Sm^r^ transconjugants that were further screened for chloramphenicol sensitivity as described (Whipple, 1998). The *lrp* coding region was amplified with the oligonucleotides DC531f (5′-GGAATTCCATATGGTAGATAGCAAGAAGCGCCCTGG-3′) and DC1463r (5′-CCGCTCGAGTTAGCGCGTCTTAATAACCAGAGC-3′) as primers and genomic DNA of strain FW102 as template. The purified NdeI plus XhoI amplicon was ligated in similarly digested and dephosphorylated plasmid pET28a plasmid DNA and transformed in competent cells of strain DH5α. Plasmid DNA extracted from a correct clone (pET28-*lrp*) was subsequently transformed in the overexpression strain BL21 (DE3). All constructs were verified by DNA sequencing.

### Overexpression and Purification of *E. coli* Arginine Repressor and Leucine-Responsive Regulatory Protein

Untagged hexameric ArgR was purified to electrophoretic homogeneity from an IPTG (isopropyl-β-d-1-thiogalactopyranoside)-induced culture of strain JM101 transformed with plasmid pDB169 as described (Lim et al., 1987). Dimeric C-terminal hexa-histidine tagged Lrp was purified to electrophoretic homogeneity from a 300 ml culture of strain BL21 (DE3) transformed with plasmid pET28-*lrp* grown on LB medium supplemented with 30 μg ml kanamycin till OD_600_ of 0.9, then induced with 1.0 mM IPTG and further grown overnight. Harvested cells were disrupted by sonication and His-tagged Lrp (hereafter called Lrp for simplicity) purified from the cleared extract by affinity chromatography on a nickel-sepharose column (HisTrap™ FF 1 ml, GE Healthcare) using an ÄKTA FPLC chromatography system (GE Healthcare). The column was initially equilibrated with washing buffer (20 mM phosphate buffer, 0.5 M NaCl, pH 7.4) containing 40 mM imidazole and elution performed by gradually increasing the imidazole concentration from an initial concentration of 40–500 mM. Fractions were analyzed by SDS-PAGE (Laemmli, 1970) and those containing pure Lrp were pooled, dialyzed to remove imidazole, and stored in storage buffer (10 mM Tris, 100 mM NaCl, 0.1 mM EDTA, 10 mM β-mercaptoethanol, 25% glycerol, pH 7.4) at −20°C.

### Enzyme Assays

Specific β-galactosidase activities were determined as described (Nguyen Le Minh et al., 2018) at 28°C in sonicated cell-free extracts of exponentially grown cultures (OD_600_ 0.4), with ortho-nitrophenyl β-d-galactopyranoside (ONPG) as substrate. Cells were grown in minimal medium supplemented with glucose (0.5%), thiamine (1.0 μg ml^−1^), kanamycin (30 μg ml^−1^), and when indicated supplemented with amino acids at 100 μg ml^−1^.

### *In vitro* DNA Binding Experiments

[5′-^32^P] Single-end labeled DNA fragments used in electrophoretic mobility shift assays (EMSA), and various footprinting and premodification binding interference experiments were prepared and purified as described (Nguyen Le Minh et al., 2018). DNase I, hydroxyl radical (Tullius and Dombroski, 1986) and chemical premodification binding interference experiments (missing contact probing) (Brunelle and Schleif, 1987) were performed as described (Wang et al., 1998). In gel footprinting with the [(OP)_2_-Cu^+^] ion (Kuwabara and Sigman, 1987) was performed as described in (Peeters et al., 2004). Binding assays were performed in binding buffer (10 mM Tris-HCl pH 7.4, 5 mM MgCl_2_, 250 mM KCl, 2.5 mM CaCl_2_, 0.5 mM DTT and 2.5% glycerol), when indicated supplemented with l-arginine (l-arginine monochloride), l-leucine or both, and incubated for 25 min at 37°C to reach equilibrium of complex formation.

## Results

### Leucine-Responsive Regulatory Protein Represses P*_artP_* but Not P*_artJ_* and P*_hisJ_* Activity

**Figure 2:**
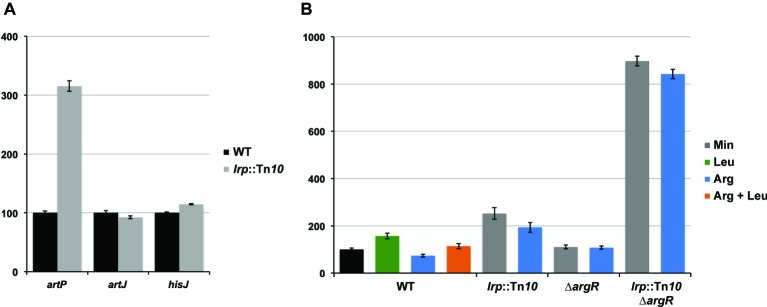
Histogram presentation of β-galactosidase specific activities. Values are the means ± standard deviation of at least three biological replicates. **(A)** Specific activities measured in cell-free extracts of *E. coli* strain FW102 (WT) and its isogenic *lrp* (*lrp*::Tn*10*) derivative bearing a single-copy *lacZ* reporter gene fusion under the control of the *artP*, *artJ*, or *hisJ* promoter/operator grown on minimal medium. 100% corresponds for each fusion construct to the activity measured in the wild type strain. **(B)** Specific activities of a single-copy *lacZ* reporter gene under the control of the *artP* promoter/operator measured in cell-free extracts of FW102 (WT) and its isogenic *lrp* (*lrp*::Tn*10*), *argR* (∆*argR*), and double *argR lrp* (*lrp*::Tn*10*/∆*argR*) mutant derivatives. Cells were grown on minimal medium, supplemented with l-arginine, l-leucine, or both, as indicated. 100% corresponds to the value obtained for the wild type strain grown on minimal medium (black colored bar).

To evaluate the potential effect of Lrp on arginine transport genes in *E. coli* we assayed gene expression with single-copy F′-borne reporter gene constructs expressing *lacZ* under the control of the P*_artP_*, P_*artJ*,_ or P*_hisJ_* promoter in cells of isogenic wild type and *lrp*::Tn*10* mutant strains grown aerobically on minimal medium and harvested in the exponential growth phase (Figure 2A). The results indicate that Lrp represses P*_artP_* activity about three-fold. In contrast, P*_artJ_* and P*_hisJ_* expression was nearly identical in absence and presence of Lrp. Hence, these two promoters and associated genes are not part of the Lrp regulon. These observations are in full agreement with the genome-wide expression profiling study (Cho et al., 2008) and validate our single copy reporter gene systems.

### Arginine Repressor and Leucine-Responsive Regulatory Protein Act as Competitive Repressors of *artP* Expression *in vivo*

Previously, we have shown that arginine-bound ArgR binds to the *artP* control region and exerts a negative effect on promoter activity (Caldara et al., 2007). To analyze the potential interplay between ArgR and Lrp in the *artP* control region, we assayed β-galactosidase activities in cell-free extracts of isogenic wild type, ∆*argR*, *lrp*::Tn*10* and double ∆*argR lrp*::Tn*10* mutants bearing a single-copy F′-borne P*_artP_*-*lacZ* reporter gene construct, grown on minimal medium and minimal medium supplemented with arginine, leucine or both (Figure 2B). The results indicate that repression by Lrp is partially counteracted by leucine supplementation. Furthermore, Lrp has a stronger negative effect than ArgR and both repressors are more potent (approximately three-fold) inhibitors of P*_artP_* activity in the absence of the other regulator [compare the ratios double mutant/∆*argR* (7.8) and *lrp*::Tn*10*/WT (2.6) on arginine for repressibility by Lrp in absence and presence of ArgR, respectively, and the ratios double mutant/*lrp*::Tn*10* (4.4) and *∆argR*/WT (1.5) on arginine for repressibility by ArgR in absence and presence of Lrp, respectively]. Therefore, we may conclude that Lrp and ArgR act as competitive repressors of P*_artP_*.

### Interferences of Leucine-Responsive Regulatory Protein and Arginine Repressor Binding to the *artP* Control Region

**Figure 3:**
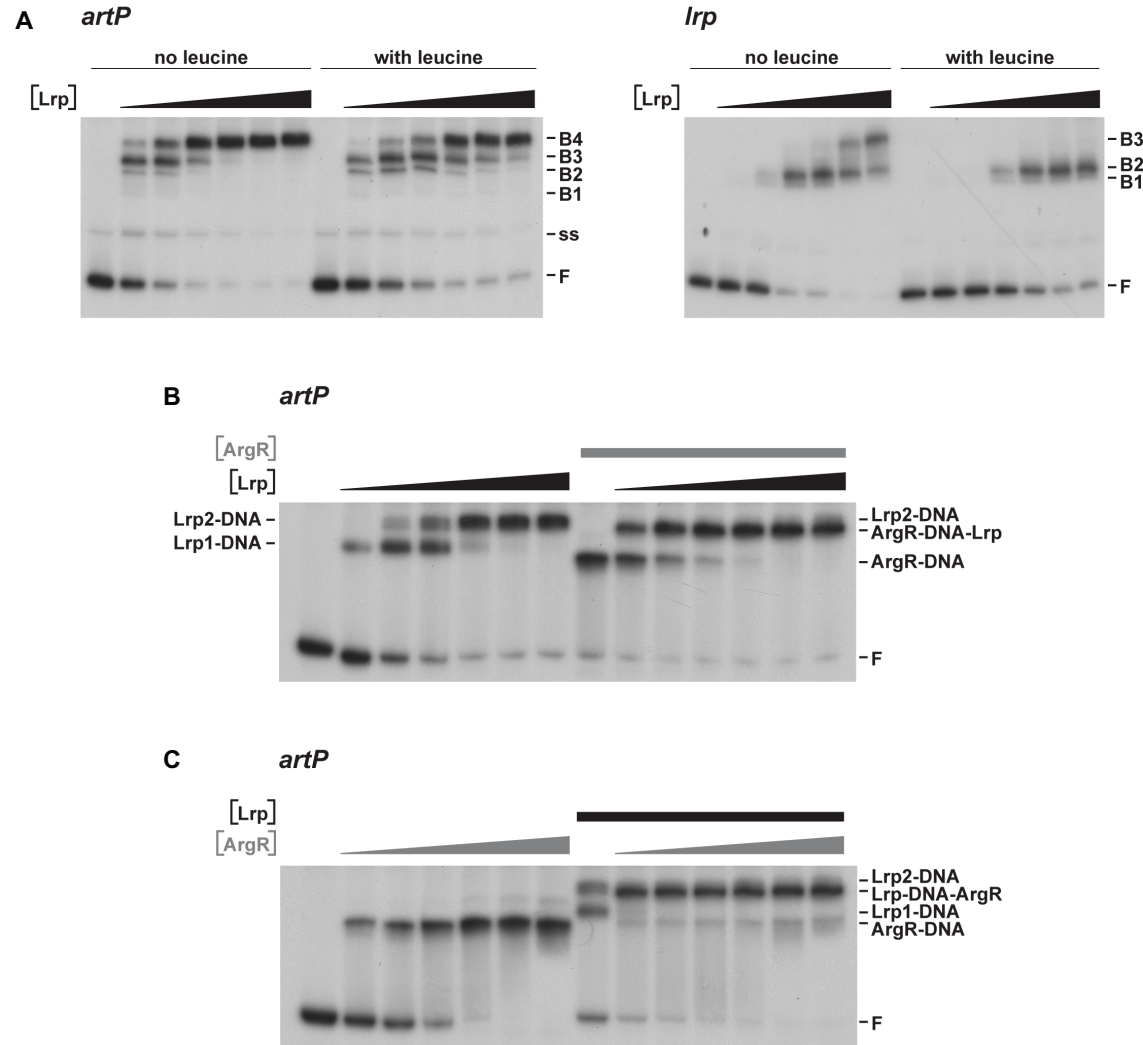
Representative autoradiographs of EMSAs with Lrp and ArgR binding to radiolabeled fragments bearing the *artP* or *lrp* control region in the presence of an excess non-labeled non-specific competitor DNA (sonicated harring sperm DNA). **(A)** Binding of Lrp to the *artP* and *lrp* control region in the absence and in the presence of 7.0 mM l-leucine. The position of free DNA (F), single-stranded DNA (ss) and the various Lrp-DNA complexes (B1 to B4) is indicated. The black triangle represents increasing Lrp concentrations corresponding to 0, 0.23, 0.46, 0.92, 1.84, 3.68, and 7.36 μM (expressed in monomer equivalents). **(B)** Binding of Lrp and Lrp plus ArgR to the *artP* control region. In the right-hand part of the panel a constant concentration of ArgR (0.48 nM, expressed in hexamer equivalents and indicated with a gray colored bar) was allowed to bind to the *artP* control region prior to the addition of increasing concentrations of Lrp (0, 0.23, 0.46, 0.92, 1.84, 3.68, and 7.36 μM indicated with a black colored triangle). The left-hand part of the panel represents binding of the same concentrations of Lrp only, as a control (notice that complexes indicated here as Lrp1 and Lrp2 correspond to complexes B3 and B4 of panel A). **(C)** In the right-hand part of the panel a constant concentration of Lrp (3.04 μM, indicated with a black colored bar) was allowed to bind to the *artP* control region prior to the addition of increasing concentrations of ArgR (0, 0.48, 0.96, 1.92, 3.84, 7.68, and 15.3 nM as indicated with a gray colored triangle). The left-hand part of the panel represents binding of the same concentrations of ArgR only, as a control.

To further unravel the molecular basis of ArgR- and Lrp-mediated repression of transcription initiation at P*_artP_*, and their interplay, we performed electrophoretic mobility shift assays (EMSAs) with purified Lrp and ArgR, alone and in combination (Figure 3). EMSAs with Lrp binding to the *artP* control region [apparent overall equilibrium dissociation constant (*K*_d_) of approximately 200 nM] revealed the concentration-dependent formation of four complexes with a different migration velocity: two minor complexes (B1 and B2), and two more abundant complexes (B3 and B4) present at a variable ratio dependent on the Lrp concentration, with higher protein concentrations favoring the formation of the slowest migrating complex B4 at the expense of all other complexes (Figure 3A). Leucine had a negative effect on total complex formation and affected the relative proportions of the different complexes, indicating that leucine lowers the overall affinity and affects the cooperativity in the binding (Figure 3A). Binding of Lrp to its own control region (autoregulation) performed as a control resulted in the formation of one minor and two more abundant complexes (Figure 3A). Again, leucine had a negative effect on binding affinity and affected the relative abundances of the various complexes. This is reminiscent of Lrp binding to its own control region in *Salmonella enterica* serovar Typhimurium (McFarland and Dorman, 2008) but is in contrast with older observations of *E. coli* Lrp binding to its own control region, which was claimed to be leucine-insensitive (Lin et al., 1992; Wang et al., 1994). *K*_d_’s for Lrp binding to various targets vary widely but are frequently in the 50–100 nM range, with a very high affinity (8 nM) observed for binding to the six binding sites in the *ilvIH* control region (Wang and Calvo, 1993) and a more similar *K*_d_ (50 nM) for binding to the *argO* gene for arginine export (Peeters et al., 2009). A large variability in binding affinities for Lrp is not surprising, since the protein exerts various functions, as a structural nucleoid associated protein (NAP) and as a more specific transcription regulator or co-regulator with about 314 binding sites on the *E. coli* genome identified in the absence of effector molecules (Shimada et al., 2015). Finally, we performed EMSAs with both ArgR and Lrp binding to the *artP* control region. When a constant amount of ArgR (0.48 nM) was allowed to bind prior to the addition of increasing concentrations of Lrp, the concentration-dependent formation of a new complex (indicated as ArgR-DNA-Lrp) migrating more slowly than the ArgR only complex and in between the Lrp1 and Lrp2 only complexes was observed (Figure 3B). This result indicates that hexameric ArgR and at least one Lrp dimer may bind simultaneously to the *artP* control region. When inversely, a constant amount of Lrp (300 nM) was allowed to bind to the *artP* control region prior to the addition of increasing concentrations of ArgR this resulted in the formation of a new complex (Lrp-DNA-ArgR) that migrates slightly below the Lrp2-DNA complex, but more slowly than the ArgR-DNA and Lrp1-DNA complexes (Figure 3C). This result suggests that ArgR is able to displace Lrp from at least one of its binding sites (or at least to bind preferentially after dissociation of Lrp), likely overlapping the ArgR binding site.

**Figure 4:**
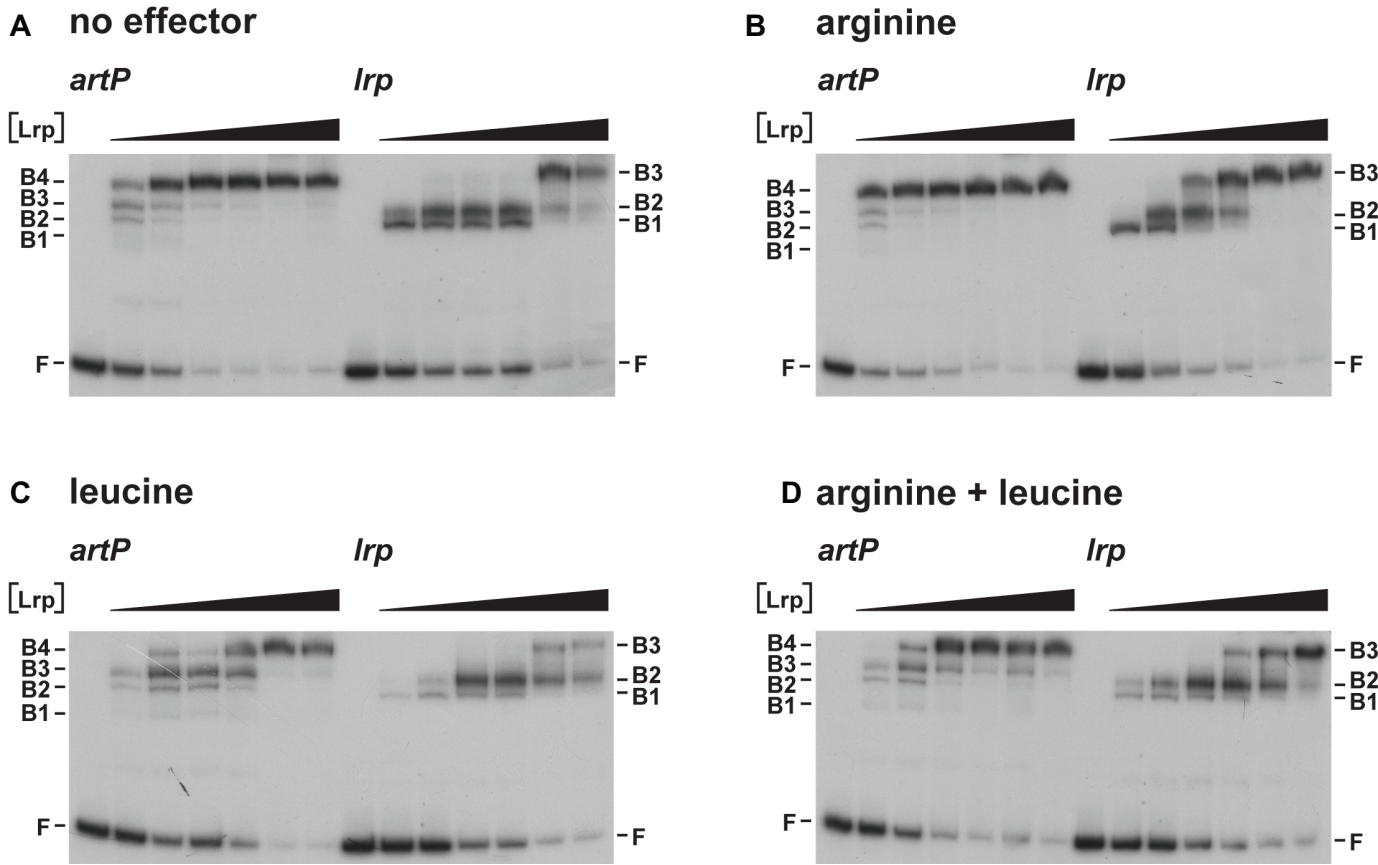
Representative autoradiographs of Lrp binding to the *artP* and *lrp* control regions in the absence **(A)** and in the presence of 10 mM l-arginine **(B)**, 10 mM l-leucine **(C)** or 10 mM of both **(D)**, as indicated. Lrp concentrations (black colored triangle) correspond to 0, 0.23, 0.46, 0.92, 1.84, 3.68, and 7.36 μM (expressed in monomer equivalents). The position of the free DNA fragments **(F)** and the Lrp-bound DNA fragments (B1, B2, B3) are indicated.

As Lrp is known to use several amino acids besides leucine as effector molecules (Hart and Blumenthal, 2011), we also tested the effect of arginine and arginine plus leucine on Lrp binding to the *artP* and *lrp* control regions in EMSA (Figure 4). The results indicate that arginine has a small stimulating effect on Lrp binding to its own control region, favoring the formation of the higher order complexes at lower protein concentrations than in the absence of effector (Figures 4A,B). Furthermore, we confirmed that leucine has a negative effect on Lrp binding to both the *artP* and *lrp* control regions (Figure 4C) and that in the presence of equimolar concentrations of both amino acids the negative effect of leucine prevails (Figure 4D). To the best of our knowledge, this is the first report of a positive effect of arginine on *E. coli* Lrp binding, but the Lrp ortholog of *Mycobacterium tuberculosis* is known to bind arginine and several other amino acids several amino acids (Shrivastava and Ramachandran, 2007).

### Identification of Leucine-Responsive Regulatory Protein Binding Sites in the *artP* Control Region

**Figure 5:**
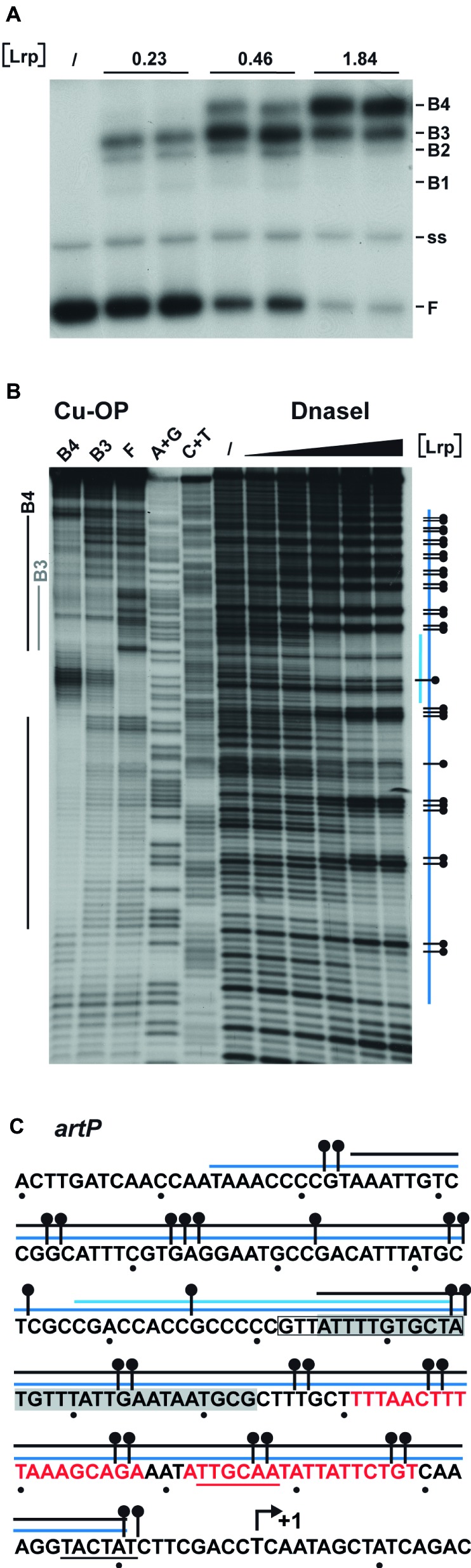
DNAse I and in gel copper-phenanthroline (OP-Cu) footprinting of Lrp binding to the *artP* control region (top or coding strand revealed). **(A)** EMSA with Lrp binding to the *artP* control region used for the in gel footprinting with the [(OP)_2_-Cu^+^] ion. Lrp concentrations (in μM) are expressed in monomer equivalents. The position of free DNA (F), single stranded DNA (ss) and the various complexes with a different migration velocity (B1 to B4) are indicated. **(B)** Autoradiograph of in gel footprinting of free DNA (F) and the major Lrp-DNA complexes B3 and B4 with a different migration velocity extracted from the gel presented in panel **(A)** after treatment with the [(OP)_2_-Cu^+^] ion and separation of the reaction products by gel electrophoresis in denaturing conditions. Regions of protection in the different complexes are indicated with gray and black colored lines for the complexes B3 and B4, respectively. A + G and C + T represent the Maxam-Gilbert sequencing ladders. In DNase I footprinting the black triangle on top of the autoradiograph represents increasing Lrp concentrations corresponding to 0, 0.46, 0.92, 1.86, 3.68, and 7.36 μM. Regions protected against DNase I cleavage are indicated with a blue colored line. The short lightblue colored line represents the region that is already protected at the lower Lrp concentrations (1.86 μM, nucleation site), the long blue colored line represents the extended zone of protection observed at higher Lrp concentrations. A short horizontal bar with a dot represents a site that becomes hyperreactive to DNase I cleavage upon Lrp binding. **(C)** Sequence of the *artP* control region (coding strand) with indication of regions of protection against enzymatic and chemical cleavage. The region of protection in complex B3 is gray shaded. Other symbols are as in panel B. The −10 and − 35 promoter elements are underlined. A short arrow represents the start of transcription (+1). The *in silico* predicted Lrp binding site (−85 to −98) (Cho et al., 2008) is boxed. Red colored letters represent ARG box sequences.

The Lrp binding sites in the *artP* control region were identified by DNase I footprinting and in gel footprinting with the [(OP)_2_-Cu^+^] ion of the major complexes (B3 and B4) with a different stoichiometry separated by EMSA (Figure 5A). DNase I footprinting indicates a concentration-dependent protection pattern (Figures 5B,C). At the lower Lrp concentrations used, a 27-nt long stretch of the coding strand (from −86 to −112 with respect to the transcription initiation site) was clearly protected against digestion by the nuclease. At higher Lrp concentrations, this zone extended further both upstream and downstream of this nucleation site (from −165 to approximately −10), with concomitant appearance of numerous regularly spaced sites of hyperreactivity, indicative of pronounced DNA deformations resulting in local minor groove widening (Lazarovici et al., 2013). In gel footprinting with the [(OP)_2_-Cu^+^] ion on the major complexes (B3, B4) with a different stoichiometry separated by gel electrophoresis (Figure 5A) revealed a 29-nt long zone of protection (from −67 to −95) on the coding strand for the faster migrating complex B3 (Figures 5B,C). This zone partially overlaps the nucleation site observed at low Lrp concentrations in DNase I footprinting. In the slower migrating complex B4, the region of protection is extended on both sides and an additional zone of clear protection is visible between positions −117 and −156. Interestingly, the two zones of clear protection are separated by an approximately 20-bp long G + C rich stretch (−96 to −116, 76% G + C) showing strong hyperreactivity. In complex B4, protection in the downstream direction extends to approximately position −10, which corresponds roughly to the border of protection observed in DNase I footprinting. The extended zones of protection indicate an overlap with the previously identified binding site for ArgR (Figure 5C; Caldara et al., 2007), which is compatible with the binding interference of the two regulators as observed in mixed EMSAs (Figure 3) and the competitive repression detected *in vivo* (Figure 2B).

### Identification of Arginine Repressor-DNA Contacts in the *lrp* Control Region

**Figure 6:**
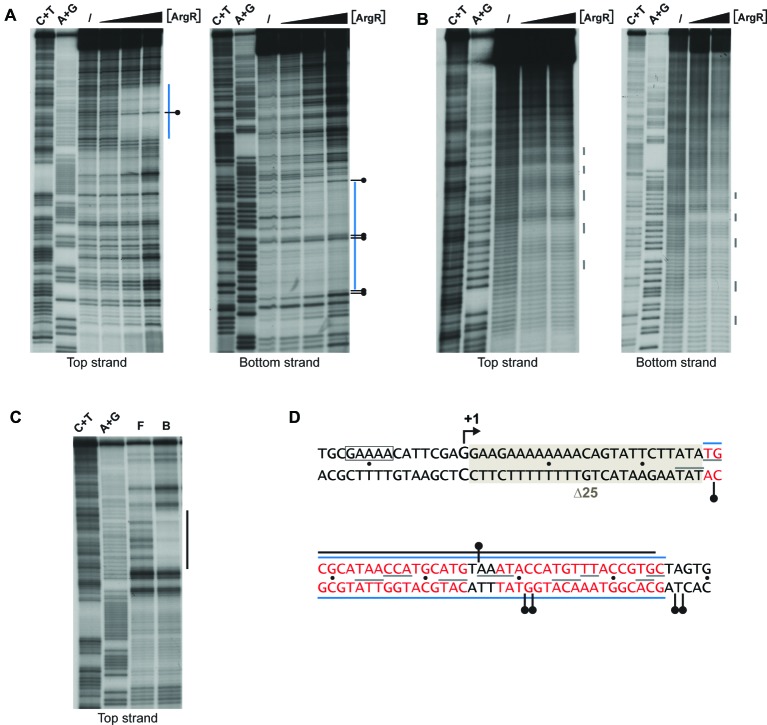
Enzymatic and chemical footprinting of ArgR binding to the *lrp* control region. **(A)** DNase I footprinting. A blue bar represents the zone of protection. A short horizontal bar with a dot represents a site that becomes hyperreactive to DNase I cleavage upon ArgR binding. ArgR concentrations used are 0, 0.39, 3.91, and 39.1 nM. C + T and A + G correspond to the Maxam-Gilbert sequencing reactions. **(B)** Hydroxyl radical footprinting. ArgR concentrations used are 0, 39.1 and 78.3 nM. Short gray colored vertical lines represent sites of protection. **(C)** In gel footprinting with the [(OP)_2_-Cu^+^] ion. F and B correspond to free and ArgR-bound DNA recovered from gel after treatment with the [(OP)_2_-Cu^+^] ion. **(D)** Nucleotide sequence with indication of zones of protection. Symbols are as in panels A, B and C. Red colored letters represent the 18 bp ARG boxes. The −10 promoter element is boxed. The 25-bp deletion of mutant ∆25 used for reporter gene assays (Figure 8) is gray shaded. The Lrp binding site as defined by DNase I footprinting (Wang et al., 1994) extends from position −106 to −20 and is not included in this figure.

**Figure 7:**
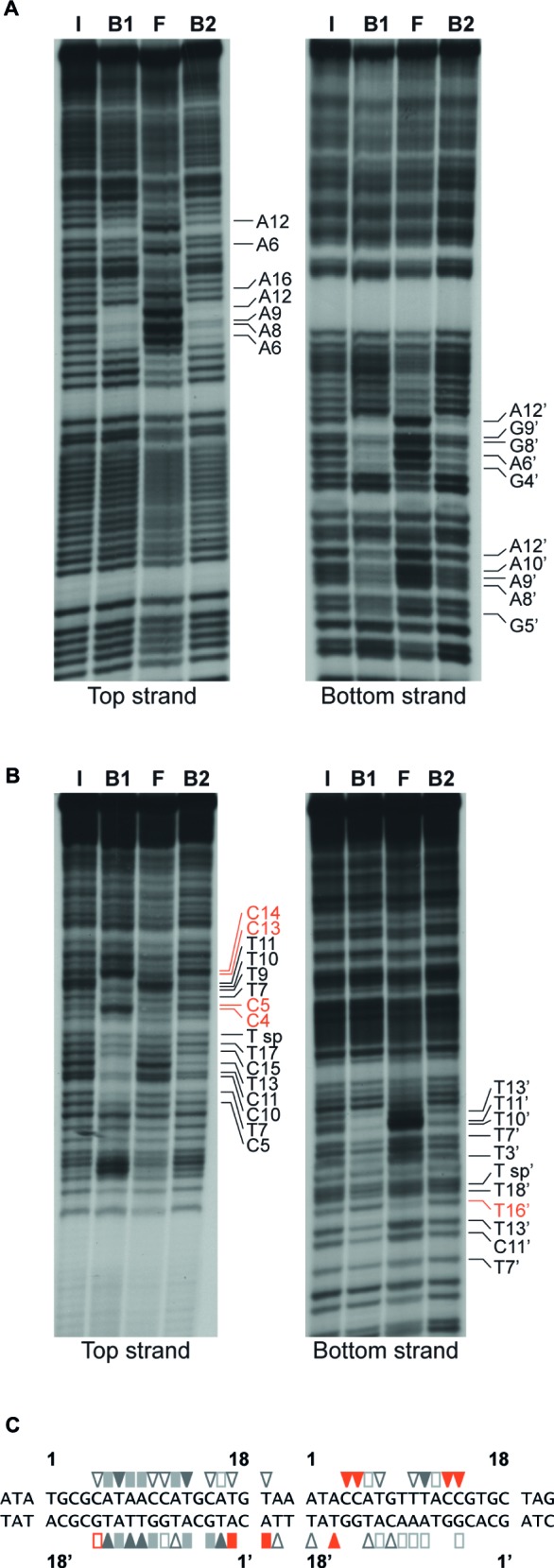
Missing contact probing with ArgR binding to the *lrp* control region. **(A)** Depurination (citrate) and **(B)** depyrimidination (hydrazine). Sparingly modified DNA molecules incubated in the absence (I = input) and in the presence of various concentrations of ArgR, resulting in approximately 50% binding for F1 and B1, and > 90% for F2 and B2, were separated on basis of their affinity for the protein by gel electrophoresis in native conditions, extracted from gel, cleaved at the positions of modification with piperidine and equal amounts of the reaction products separated by gel electrophoresis in denaturing conditions. Positions that upon base removal interfere strongly or moderately are indicated. Positions that upon removal result in better binding are indicated with orange colored letters **(C)** Nucleotide sequence of the ArgR binding site in the *lrp* control region with indication of positions that upon modification interfere strongly (filled symbols) or moderately (open symbols) with ArgR binding are indicated. Orange colored symbols represent inverse effects. ARG box sequences separated by a 3 bp spacer are numbered 1 to 18 for the top (coding) strand and 1′ to 18′ for the bottom strand in the 5′-3′ direction.

Previously it was shown that ArgR binds to the *lrp* control region *in vivo* (Cho et al., 2011, 2015). Here, we perform a detailed *in vitro* interaction study of purified ArgR binding to the *lrp* control region by a combination of enzymatic and chemical footprinting techniques (DNase I, hydroxyl radical, “in-gel” footprinting with the [(OP)_2_-Cu^+^] ion), and establish a high-resolution base-specific contact map based on premodification binding interference assays (Figures 6, 7). DNase I revealed the protection of a 37-nt long stretch on both stands (Figures 6A,D) extending from position +29 to +65 downstream of the transcription initiation site. This zone of protection comprises two 18-bp imperfect palindromes separated by 3 bp, a canonical configuration for an *E. coli* ArgR binding site associated with arginine biosynthetic and transport genes (Figure 6D; Charlier et al., 1992; Tian et al., 1992; Caldara et al., 2007). Hyperreactivity for DNase I interrupting the zone of protection and in the adjacent flanking regions is indicative of DNA bending associated with minor groove widening. In gel footprinting of the single ArgR-DNA complex separated from free DNA by gel electrophoresis with the [(OP)_2_-Cu^+^] ion confirmed the position of this zone of interaction (Figures 6C,D) that also corresponds to the *in vivo* protected target (Cho et al., 2015). Chemical footprinting with the very small and highly reactive hydroxyl radical (Tullius and Dombroski, 1986), allowed to break up this global region of protection into five small subzones on both strands, each one being 2 to 4 nt long, with the centers of protection separated by approximately one full helical turn and slightly shifted toward the 3′-end on complementary strands (Figures 6B,D). Such a pattern is indicative of ArgR binding to one face of the DNA helix (Oackley and Dervan, 1990), as observed previously with ArgR binding to the ARG boxes of arginine biosynthetic genes (Charlier et al., 1992; Wang et al., 1998). The position of the ArgR binding site in the *lrp* control region, downstream of the transcription start, is unusual for strong ArgR-repressed promoters since in arginine biosynthetic operators the ARG boxes are located upstream of the transcription start and partially overlap the −10 and/or − 35 promoter elements (Charlier et al., 1992; Tian et al., 1992; Caldara et al., 2007).

Base-specific contacts of ArgR with purine and pyrimidine bases in the *lrp* control region were identified with chemical premodification binding interference experiments (Figure 7; Brunelle and Schleif, 1987). Sparingly modified 5′-single-end-labeled DNA (treated with either citrate at pH 4.0 and 80°C or hydrazine at 25°C for purine and pyrimidine missing contact probing, respectively) was incubated with various concentrations of ArgR and separated on basis of affinity for the regulator in a native EMSA. Free and ArgR-bound DNA molecules were subsequently extracted from the gel, cleaved at the position of modification by piperidine treatment, and the reaction products analyzed by gel electrophoresis in denaturing conditions. In such an experiment, molecules modified at a position important for complex formation, and thus exhibiting a reduced affinity for the repressor, are expected to be overrepresented in the free DNA and underrepresented in the bound form, whereas molecules with a modification at a position that does not significantly contribute to complex formation are expected to be equally distributed over free and bound forms. The results indicate that all strong and moderate negative effects of base removal on ArgR binding are located within the ARG boxes as defined above (Figure 7). In the promoter-proximal ARG box, the strongest negative effects were observed upon removal of A_6_, T_7_, A_8_, A_9_, A_12_, and T_13_ from the top strand (coding strand) and T_3’_, G_4’_, A_6’_, G_9’_, T_10’_, T_11’_, A_12’_, and T_13’_ of the bottom strand. In the promoter-distal box, only removal of T_11_ occasioned a strong negative effect on ArgR binding. This is in full agreement with the more degenerate character of this ARG box compared to the consensus sequence (Figure 7C; Charlier et al., 1992). Of the strong interfering positions, A_6_, T_7_, A_12_, T_13_ and the symmetrical counterparts are among the most conserved positions in ARG box sequences previously described, and their importance in complex formation and gene regulation has been demonstrated for various genes and operons of the ArgR regulon (Charlier et al., 1992; Wang et al., 1998; Caldara et al., 2007). In various instances (C_4_, C_5_, C_13_, and C_14_ of the top strand and T_16’_ of the bottom strand), base removal in the downstream ARG box of the *lrp* control region resulted in enhanced ArgR binding. Some of these inverse signals are generated upon removal of bases occupying highly conserved positions among ARG boxes (especially positions 4 and 13) but do not correspond to the consensus sequence (G_4_ and T_13_, respectively) in the *lrp* operator. These results indicate that the presence of a non-consensus base, showing a different distribution of base-specific groups as potential donors and acceptors in hydrogen bond formation in the major groove segment contacted by ArgR, at these positions in the *lrp* control region does not contribute to ArgR binding but instead generates a steric hindrance for the establishment of another, nearby contact (see “Discussion”).

### Effects of Arginine Repressor and Leucine-Responsive Regulatory Protein on *in vivo lrp* Gene Expression

**Figure 8:**
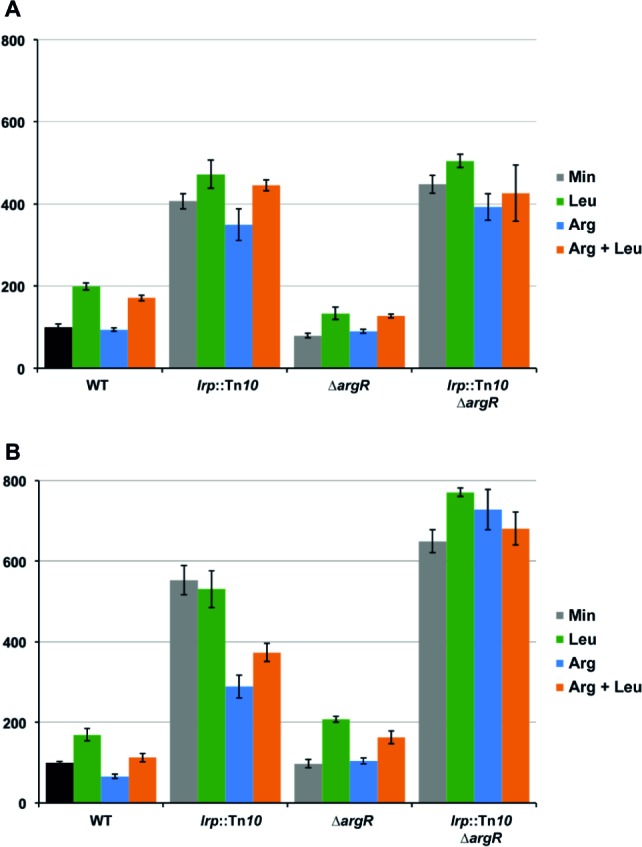
Histogram presentation of β-galactosidase specific activities measured in cell-free extracts of *E. coli* strain FW102 (WT) and its isogenic *lrp* (*lrp*::Tn*10*), *argR* (∆*argR*) and double *lrp argR* (*lrp*::Tn*10* ∆*argR*) mutants bearing the wild type *lrp* promoter/operator-*lacZ* **(A)** or mutant *lrp*∆25 promoter/operator-*lac*Z fusion **(B)** on a single-copy F’ episome. Cells were grown in minimal medium, when indicated supplemented with l-arginine, l-leucine, or both. Values are the means ± standard deviation of at least three biological replicates. 100% corresponds to the activity measured for each fusion construct introduced in the WT strain and grown on minimal medium (black colored bar).

The effect of the transcriptional regulators ArgR and Lrp on *lrp* gene expression was determined with a single-copy F′-borne reporter gene construct expressing *lacZ* under the control of the P*_lrp_* promoter in isogenic wild type, ∆*argR*, *lrp*::Tn*10* and double ∆*argR lrp*::Tn*10* mutant strains grown on minimal medium and minimal medium supplemented with arginine, leucine or both. The results (Figure 8A) indicate negative autoregulation (about four-fold, compare WT and *lrp*::Tn*10* on minimal medium) that is partially counteracted by leucine as effector molecule (about two-fold, compare WT minimal and leucine). This reciprocal mode of autoregulation has also been proposed for *lrp* in *Salmonella typhimurium* (McFarland and Dorman, 2008), but is in contradiction with previous work indicating leucine-independent negative regulation of *E. coli lrp* (Lin et al., 1992; Wang et al., 1994). Besides autoregulation, we observed little or no negative effect of ArgR and arginine on *lrp* promoter activity. Instead, slightly lower values (about 1.3-fold) were measured in the ∆*argR* mutant compared to the WT. This reduction might be due to a stronger repression exerted by Lrp in the absence of any interfering ArgR binding [5.6-fold (ratio double mutant/∆*argR*) compared to 3.7-fold in the presence of liganded ArgR (ratio *lrp*::Tn*10*/WT grown on arginine)]. As shown above, the ARG boxes in the *lrp* control region are located downstream of the transcription start site. In this context, it is worth noticing that ArgR binding to the ARG boxes overlapping the *E. coli car*P2 promoter strongly represses transcription initiation from this promoter but does not hinder transcription initiated 67 nt more upstream at the *car*P1 promoter (Charlier et al., 1988, 2018). Hence, it appears that ArgR is unable to function as an efficient roadblock for an elongating RNA polymerase. To further verify this hypothesis, we constructed a 25 bp deletion mutant (∆25) of the *lrp* operator-*lacZ* fusion construct in which the ARG boxes are positioned immediately downstream of the transcription initiation site (Figure 6D). Reporter gene assays performed with this construct indicate that the deletion has a negative effect on intrinsic promoter strength [about 1.7-fold, compare WT and mutant operator in the absence of regulation (double ∆*argR lrp*::Tn*10* background)] (Figures 8A,B). In the WT background arginine has a slight negative (1.7-fold) and leucine a slight positive effect (1.7-fold) on the ∆25 mutant promoter activity. This arginine-dependent reduction is indicative of a weak ArgR-mediated repression, which is, however, not reflected in the activities measured in the ∆*argR* background. This might again be due to a stronger repression exerted by Lrp in the absence of interfering ArgR binding. Indeed, repression by Lrp is about 1.5-fold stronger in the absence of ArgR than in its presence [compare the 6.6 to 7.0-fold repression in the absence (ratio double mutant/∆*argR* on minimal or arginine supplemented medium, respectively) with the 4.4-fold repression in the presence of liganded ArgR (ratio *lrp*::Tn*10*/WT on arginine supplemented medium)]. In agreement with this observation repression by liganded ArgR is also stronger in the absence of Lrp (2.5-fold, ratio double mutant/*lrp*::Tn*10* grown both on arginine) than in its presence.

## Discussion

Independent studies using different approaches have shown that transcription of the *artPIQM* operon is negatively regulated by ArgR (Caldara et al., 2006, 2007; Cho et al., 2015) and Lrp (Cho et al., 2008; Shimada et al., 2015). Arginine as an effector molecule of ArgR is known to enhance its negative effect (corepressor) on P*_artP_*, whereas leucine partially counteracts the negative effect of Lrp (reciprocal regulation mode) and thus acts as an inducer. Many synthesis, degradation and transport systems for amino acids and amino acid sensing transcription factors are subjected to transcriptional regulation by Lrp (Cho et al., 2011; Shimada et al., 2015). Several amino acid uptake and export systems are either negatively or positively regulated by Lrp and leucine in a reciprocal manner, whereby leucine attenuates the effect of Lrp. This is for instance the case for serine (*sdaC*), alanine (*cycA*), proline (*proY*), leucine (*leuE*), and also arginine export (*argO*) (Kutukova et al., 2005; Cho et al., 2008; Peeters et al., 2009). In contrast, Lrp-mediated regulation of several transporters for aromatic amino acids occurs according to the concerted mode, whereby leucine potentiates the negative effect of the regulator (Cho et al., 2008). As a consequence of the widespread effect of Lrp on amino acid metabolism, amino acid pools in isogenic WT and *lrp* mutant strains may vary to some extent (Shimada et al., 2015), which may in turn affect the action of amino acid sensing transcription factors, especially upon growth in minimal or leucine supplemented medium.

Many bacterial promoters are regulated by multiple transcription factors for the integration of distinct environmental signals (Paul et al., 2007; Ogasawara et al., 2010). Their positive or negative interplay in the control of one or more promoters illustrates just one aspect of the complexity, diversity and versatility of bacterial gene regulation mechanisms, which are frequently multi-layered and operate at different levels and stages of gene expression (for an overview, see Bervoets and Charlier, 2019). Here, we analyzed the interplay between ArgR and Lrp in the control of P*_artP_* activity *in vivo* and *in vitro* and demonstrate that each repressor is a more potent inhibitor of P*_artP_* activity in the absence of the other. Hence, they act as competitive repressors. This observation is in agreement with, and may be explained by, the existence of partially overlapping binding sites for ArgR and Lrp in the *artP* control region. Binding interferences indicate that even though ArgR and Lrp may bind simultaneously to the *artP* control region, the number of Lrp dimers that can bind in the presence of ArgR is reduced, and thus the formation of higher order Lrp-DNA complexes is inhibited. This may be explained by the observation that the nucleation site for Lrp binding does not overlap the ARG boxes, whereas the more extended zone of interaction identified at higher Lrp concentrations does overlap (Figure 5C). The concentration-dependent formation of four complexes and the effect of leucine on binding affinity and cooperativity is reminiscent of Lrp binding to several other Lrp-regulated genes and artificial operator constructs (Azam and Ishihama, 1999), among which the *E. coli argO* gene for arginine export, that is activated in a competitive manner by Lrp and ArgP (a LysR-type transcriptional regulator) (Peeters et al., 2009; Nguyen Le Minh et al., 2018). Our findings are also fully compatible with the results of a detailed analysis of cooperative Lrp binding to various targets, indicating that leucine decreases the intrinsic affinity of Lrp for a single binding site but enhances the cooperativity in the binding to multiple sites (in a spacer length dependent manner) (Chen et al., 2005).

At low concentrations (nM range) Lrp exists primarily as a homodimer in solution (Chen et al., 2001; Chen and Calvo, 2002), but the protein can form higher oligomeric structures at higher concentrations *in vitro*, upon DNA binding, and *in vivo* (where it is present at μM concentrations), with leucine favoring the dissociation of hexadecameric Lrp into two leucine-bound octamers (Chen et al., 2005). In view of this effect of leucine on the self-association of Lrp, it has been postulated that the hexadecameric form would be the active one for promoters that are either activated or repressed by Lrp and leucine in the reciprocal mode, whereas the leucine-bound octamer would be the active form for promoters that are regulated in the concerted manner by the regulator and its effector (Chen et al., 2001). Our results suggest the cooperative binding of at least four Lrp dimers to the *artP* control region, although we cannot exclude the existence of some higher oligomeric forms of the protein in solution. The consensus Lrp binding site [CAG-N9(A + T rich)-CTG] proposed on basis of ChIP-chip experiments (Cho et al., 2008) is rather degenerated and various slightly different consensus sites have been proposed in the past (Cui et al., 1996; Shultzaberger and Schneider, 1999; Peterson et al., 2007). This is not surprising, since Lrp, as a nucleoid associated protein (NAP), also plays an important structural role. The nucleation site identified by different footprinting methods in the *artP* control region bears the motif G-N_2–3_-TTT, which is also found in the six well-characterized Lrp binding sites in the *E. coli pap* control region as part of the consensus (Nou et al., 1995). Several stretches showing imperfect conservation of this motif can also be found in the regions flanking the nucleation site that are protected in the higher oligomeric Lrp-DNA complexes (in gel footprinting) and at higher Lrp concentrations (DNase I) (Figure 5). Furthermore, a unique potential Lrp binding site predicted on basis of *in silico* analyses (Cho et al., 2008) is comprised within the nucleation site (Figure 5C). The extended and regularly spaced hyperreactivity for DNase I cleavage observed in complexes made at higher Lrp concentrations are indicative of pronounced DNA deformations (Figure 5B), an observation that is compatible with DNA wrapping around octameric Lrp as in the Lrp-DNA cocrystal structure (de los Rios and Perona, 2007). It is therefore likely that individual DNA-bound Lrp dimers interact to form a higher oligomeric assembly in which the *artP* operator DNA is highly deformed.

The interference of Lrp binding with the ArgR-specific control of P*_artP_* activity may also explain to a large extent the major differences observed in the *in vivo* repressibility of *artJ* and *artPIQM* transcription (Caldara et al., 2006, 2007) and the higher degree of *in vivo* occupancy by ArgR of the *artJ* operator compared to *artP* (Cho et al., 2011). Indeed, P*_artJ_* is about nine-fold more repressible by liganded ArgR than P*_artP_* even though *in vitro* both control regions exhibit a very similar affinity for purified ArgR and an identical degree of overlap of the ARG boxes with the promoter elements (Caldara et al., 2007). In this context, it is worth noticing that in different growth conditions, and besides Lrp, additional regulatory molecules may influence *artP* transcription and its ArgR-specific regulation *in vivo*. In a genomic SELEX experiment the *artP* control region was also identified as a target site for AllR (Hasegawa et al., 2008), a member of the large IclR family of transcription factors that controls a set of genes for degradation and reutilization of purines. It senses both allantoin and glyoxylate (Walker et al., 2006). However, potential binding site(s) for AllR in the *artP* operator have not been identified and an effect of AllR on P_artP_ has not been demonstrated. Furthermore, *artP* mRNA levels were found to be about two-fold upregulated under stressful conditions, including the presence of the superoxide generating compound paraquat, and the weak acid salt sodium salicylate (Pomposiello et al., 2001). Furthermore, upregulation of the *artP* mRNA level was also observed at the onset of stationary phase growth, likely by initiation from additional Eσ^S^-specific promoters located upstream of the major P*_artP_* studied here (Lacour and Landini, 2004). This all appears now to be linked to the effect of the small regulatory RNA (sRNA) SdsR that is transcribed by Eσ^S^ and accumulates in stationary phase (Fröhlich et al., 2012). SdsR is highly conserved among enterobacteriaceae and was shown to directly affect *artPIQM* expression in *Salmonella* in a Hfq-dependent manner (Fröhlich et al., 2016).

ArgR binds to two 18 bp ARG boxes located downstream of the transcription initiation site (Figure 6), an unusual position for an ArgR binding site, at least in the control region of highly repressible genes and operons, where they overlap the promoter (Charlier et al., 1992; Tian et al., 1992; Caldara et al., 2007). High-resolution contact probing revealed base specific details of ArgR-DNA contacts, and indicates that the promoter-proximal ARG box contributes more to the energy of complex formation than the promoter distal box (Figure 7). Inverse effects (higher affinity binding) upon removal of particular bases in this promoter distal ARG box was observed as well and was particularly striking for the cytosine residues in position 4, 5, 13 and 14 of the top strand (Figures 7B,C). The removal of these cytosine residues appears to eliminate a hindrance for the establishment of nearby contacts. Position 4 and 13 are highly conserved and generally consist of a G-C and T-A pair, respectively, and are located in operator segments where the major groove is facing the repressor (Wang et al., 1998). Since the removal of the complementary guanine residues of the bottom strand did not show such an inverse effect, we may hypothesize that the negative effect on ArgR binding of C-G base pairs at positions 4 and 13 is due to the presence of the exocyclic amino group of the cytosine ring pointing into the major groove. Such a group, which may serve as a donor in the formation of a hydrogen bond, is neither present on the G_4_ and T_13_ residues found at these positions on the top strand of the consensus sequence. Instead, G and T carry a carbonyl group pointing in the major groove, which may function as an acceptor in the formation of a hydrogen bond.

Our reporter gene assays point to an indirect effect of ArgR on *lrp* expression through interference with negative autoregulation (Figure 8). In this context, it is worth noticing that the intracellular concentration of free arginine in *E. coli* cells grown on minimal medium (0.14 mM; Caldara et al., 2008) is already responsible for significant *in vivo* repression (Charlier and Glansdorff, 2004; Caldara et al., 2006) and ArgR binding (Cho et al., 2015), and that the ARG boxes of the *lrp* control region are located at an unusual position downstream of the transcription initiation site (Figure 7). A stronger effect of arginine supplementation on P*_lrp_* activity was measured in a 25 bp deletion mutant in which the ARG boxes are positioned immediately downstream of the start of transcription. Previously we have shown that ArgR binding in overlap with the *car*P2 promoter elements strongly inhibits transcription initiation from this promoter but does not interfere with transcription initiated 67 nt upstream, at the *car*P1 promoter (Charlier et al., 1988). Combined, these observations suggest that ArgR is unable to function as an efficient roadblock, as some other repressors including PurR do at specific promoters (He and Zalkin, 1992). Interestingly, an indirect effect of ArgR on gene expression exerted from a far upstream binding site has been demonstrated for the *gltBDF* operon encoding one of the two major pathways for ammonia assimilation in *E. coli* (Paul et al., 2007). *gltB* promoter activity is regulated by Lrp, IHF, CRP, and ArgR, and the latter mainly interferes with the activation by Lrp (Paul et al., 2007). Hence, here ArgR acts as an anti-activator rather than as a repressor. ArgR also binds upstream (17 bp) of the −35 element of the *hisJ* promoter and exerts a weak repression. However, this inhibitory effect of ArgR on promoter activity is direct as it was also observed in a pure *in vitro* transcription assay (Caldara et al., 2007). The recent genome-wide *in vivo* ArgR binding study of Cho et al. (2015) has revealed numerous additional ArgR binding sites in intergenic regions of the *E. coli* chromosome. However, how ArgR exerts a potential direct or indirect regulatory effect on adjacent promoters and how it may interfere negatively or positively with the action of other transcription factors, including NAPs, remains to be elucidated.

## Data Availability

All datasets generated for this study are included in the manuscript and/or the supplementary files.

## Author Contributions

DC, OT, and IB conceived and designed the experiments. OT, EP, and DC performed the experiments. OT, IB, and DC analyzed and interpreted the data. DC wrote the first draft of the paper. All authors contributed to manuscript revisions, read, and approved the submitted version.

### Conflict of Interest Statement

The authors declare that the research was conducted in the absence of any commercial or financial relationships that could be construed as a potential conflict of interest.
